# Technology to Produce High-Purity Anhydrous Rubidium Perrhenate on an Industrial Scale

**DOI:** 10.3390/ma12071130

**Published:** 2019-04-06

**Authors:** Katarzyna Leszczyńska-Sejda, Grzegorz Benke, Mateusz Ciszewski, Michał Drzazga

**Affiliations:** Instytut Metali Nieżelaznych (IMN), Hydrometallurgy Department, ul. Sowińskiego 5, 44-100 Gliwice, Poland; grzegorzb@imn.gliwice.pl (G.B.); mateuszc@imn.gliwice.pl (M.C.); michald@imn.gliwice.pl (M.D.)

**Keywords:** rubidium, rhenium, perrhenate, ion-exchange

## Abstract

Technology used to produce high purity anhydrous rubidium perrhenate on an industrial scale from high purity perrhenic acid and rubidium nitrate by the ion-exchange method is described in this paper. This material is dedicated to catalyst preparation, therefore, strict purity requirements have to be fulfilled. These are satisfied by combining rubidium ion sorption on an ion exchange column and the subsequent elution of the high purity perrhenic acid solution, followed by crystallization, evaporation, purification, and drying. In the current study, rubidium and rhenium contents were found to be 22.5 wt.% and 55.4 wt.%, respectively, while contaminations were as follows: <2 ppm As, <2 ppm Bi, <5 ppm Ca, <5 ppm Cu, <3 ppm Fe, <10 ppm K, <3 ppm Mg, <5 ppm Mo, <2 ppm Na, <5 ppm Pb, and <3 ppm Zn.

## 1. Introduction

Preparation methods of rubidium perrhenate are poorly described in scientific databases. In particular, there is no information on the industrial scale production of this compound. Its synthesis has so far been devoted to obtaining materials for physico-chemical property determination [1,2,3]. Preparation is generally based on the neutralization of perrhenic acid solution by rubidium carbonate or hydroxide in equimolar amounts, resulting in the precipitation of rubidium perrhenate [4,5]. Then, the product is purified and recrystallized. Rubidium perrhenate is a colorless crystal with a tetragonal bipyramidal symmetry [6,7]. Similar to potassium, cesium, and silver, perrhenates are characterized by low water solubility [2]. Their melting temperature is 871–878 K [4,5]. The ion-exchange technique proposed in this manuscript was successfully used for nickel(II) perrhenate, cobalt(II) perrhenate, chromium(III) perrhenate, and cesium perrhenate production and has been described in past works [8,9,10,11]. This method (for nickel(II) and cobalt(II)) has been implemented in industry practice. The installation to produce these compounds is located in the Instytut Metali Nieżelaznych (IMN), Gliwice, Poland [8]. In this work, the production technology of high purity anhydrous rubidium perrhenate is presented. The obtained high-purity materials may be used for the production of inorganic and organic Re compounds, like catalysts, e.g., methyltrioxorhenium (MTO), rhenium carbonyl (Re_2_(CO)_10_), and its derivatives [12,13]. It may also be an important additive to heterogeneous (e.g., Al_2_O_3_- or SiO_2_-based) catalysts [14,15,16,17]. It is important to point out that rhenium carbonyl is currently obtained from NH_4_ReO_4_, while methyltrioxorhenium is prepared from AgReO_4_ [17]. The solubility of rubidium perrhenate is similar to silver and cesium perrhenates; therefore, it may also be used for the synthesis of MTO and Re_2_(CO)_10_. The potential advantages of RbReO_4_ application may be a shorter reaction time, an enhanced reaction yield, and the availability of eco-friendly solvents [13]. Rubidium can also be used as a doping component of catalysts for ethylene oxide production [14,15,16]. Currently, this type of catalyst is produced by reactions of aqueous perrhenate and rubidium solutions [14,15,16]. Replacing these two solutions by one rubidium perrhenate solution would allow uniform migration of active species within a support matrix. Additionally, unnecessary components, like nitrate and ammonium ions, are eliminated.

## 2. Experimental Section

### 2.1. Materials

High-purity perrhenic acid, which was prepared using ion-exchange, solvent extraction, or ion-exchange methods at the IMN, was used as the source of rhenium [18,19,20,21]. Its purity was confirmed by quantitative analysis, which showed the following components: 100.0–500.0 g/dm^3^ Re, <20 ppm NH_4_^+^, <3 ppm Al, <2 ppm As, <2 ppm Bi, <3 ppm Ca, <5 ppm Co, <3 ppm Cu, <3 ppm Fe, <10 ppm K, <3 ppm Mg, <5 ppm Mo, <5 ppm Ni, <5 ppm Pb, and <3 ppm Zn. Rubidium nitrate, which was used as a rubidium source, was purchased from Alfa Aesar (Haverhill, MA, USA). Strongly acidic hydrogenated cation-exchange resins (CT169, PFC100x10, C160) were purchased from Purolite (Bala Cynwyd, PA, USA), while SP112 was purchased from Bayer Chemicals (Leverkusen, Germany). More detailed information concerning selected resins is presented in Table 1. A solution of 30% H_2_O_2_ from Merck (Darmstadt, Germany) and acetone from Sigma Aldrich (Saint Louis, MO, USA) was used for product purification. Syntheses were performed using double-distilled water (<2 μS/cm).

### 2.2. Preparation of Rubidium Perrhenate by Ion Exchange

The method used for anhydrous nanocrystalline rubidium perrhenate synthesis was based on previous work [8]. It is considered to be an alternative to the typical path of synthesis by neutralization of perrhenic acid with rubidium carbonate [7]. The mixing of two precursor solutions was replaced by a two-stage method combining rubidium sorption and its elution using perrhenic acid solution:

sorption: (cationite)−H^+^ + Rb^+^ → (cationite)−Rb^+^ + H^+^ (1)

elution: (cationite)−Rb^+^ + HReO_4_ → (cationite)−H^+^ + RbReO_4_ (2)

The investigation was carried out under both static and dynamic conditions. 

#### 2.2.1. Static Condition

##### Sorption Stage

Preliminary tests were performed under static conditions to select the best cationites for rubidium ion sorption. Then, for selected resins, the influences of different process conditions (temperature, contact time, and rubidium concentration in an initial solution) on rubidium sorption efficiency and/or the rubidium saturation degree were determined. Selection of the proper ion-exchange resin was done according to the following methodology: 10 g of resin was mixed with 0.1 dm^3^ of RbNO_3_ solution (5.0 g/dm^3^ Rb) for 30 min at ambient temperature and then vacuum-filtrated. Solutions were analyzed with respect to the rubidium content. The rubidium sorption efficiency was determined using Equation (3):

(3) $$W_{\mathrm{Rb}}=\frac{m_{0\mathrm{Rb}}{-C}_{\mathrm{Rb}}V}{m_{0\mathrm{Rb}}}\times 100\%$$

where *m*_0Rb_ is the initial mass of rubidium in a solution (g); *C*_Rb_ is the rubidium concentration in a solution after sorption (g/dm^3^); and *V* is the volume of the solution after sorption (dm^3^).

The preliminary experiments were initially performed with specific parameters and then these were extended to various time, temperature, and concentration ranges. The examined temperature range was 20–60 °C with a contact time of 30–120 min, and a rubidium concentration of 0.5–20.0 g/dm^3^. Forty-gram ionite portions were applied. The influences of contact time and rubidium concentration on sorption efficiency were investigated at ambient temperature, while the Rb concentration effect was investigated for a contact time of 30 min.

##### Elution Stage

Next, the effects of the contact time, temperature, and Re:Rb ratio on rubidium elution were examined. The rubidium content after sorption was 4.4%, and 4.2% in PFC100x10 and C160, respectively. The following methodology was applied in this part: 5 g of rubidium-bearing resin was mixed with 0.01 dm^3^ of HReO_4_ (100.0 g/dm^3^ Re). Solutions were mixed for 30 min and filtered using a vacuum pump. Parameters were examined in the following ranges: contact time of 30–180 min, temperature of 20–60 °C, and Re:Rb ratio of 5:1–20:1. The influences of temperature and the Re:Rb ratio were investigated at a contact time of 120 min. The elution efficiency was determined using Equation (4):

(4) $${W\prime }_{\mathrm{Rb}}=\frac{{C\prime }_{\mathrm{Rb}}V\prime }{m_{0\mathrm{Rb}}{-C}_{\mathrm{Rb}}V}\times 100\%$$

where *C*′_Rb_ is the rubidium concentration of a solution after elution (g/dm^3^), *V*′ is the volume of solution after elution (dm^3^), *m*_0Rb_ is the initial mass of rubidium in a solution (g), *C*_Rb_ is the rubidium concentration in a solution after sorption (g/dm^3^), and *V* is the volume of the solution after sorption (dm^3^).

#### 2.2.2. Dynamic Test

Dynamic tests were performed using the following methodology: a column of 0.025 m diameter was filled with 100 g PFC100 resin and then treated with an RbNO_3_ solution (5.0 g/dm^3^ Rb) until the concentration of rubidium in the effluent returned to the initial level. The effluent was then divided into three parts of 0.1 dm^3^, and the rubidium content was analyzed. Next, the column was washed with distilled water until pH 7.0; then, 0.6 dm^3^ of perrhenic acid (100.0 g/dm^3^ Re) was passed through it. Resin was washed until neutral pH and then reused for further processes. Solutions consisting of eluates and the first parts of washing solutions from three cycles (sorption, washing, elution) were used in after concentration. The efficiency of Rb^+^ sorption and elution as well as the saturation of resin by Rb were determined, based on Equations (3)–(5), respectively. The degree of rubidium saturation was determined using Equation (5):

(5) $$S_{\mathrm{Rb}}=\frac{m_{0\mathrm{Rb}}{-C}_{\mathrm{Rb}}V}{m_{J}}\times 100\%$$

where *m*_0Rb_ is the initial mass of rubidium mass in a solution (g); *m_J_* is the mass of ion-exchange resin (g); *C*_Rb_ is the rubidium concentration in solution after sorption (g/dm^3^); and *V* is the volume of solution after sorption (dm^3^).

#### 2.2.3. Crystalization and Purification Test

Solutions of different rubidium concentrations, i.e., one, three, six, and 9 g/dm^3^, were prepared by combining proper amounts of solutions from elution and washing after elution. Concentrated solutions were heated up to 80 °C while mixing, cooled at a cooling rate of 5 °C/min to ambient temperature, and then filtered to obtain rubidium perrhenate. Additionally, rubidium perrhenate, which was obtained using 9.0 g/dm^3^ Rb solution, was divided into five portions and purified by single or double washing. It was done using 0.05 dm^3^ of 5% or 10% H_2_O_2_ solution and/or 0.02 dm^3^ portions of anhydrous acetone. Pure crystals were dried at 80 °C until a constant mass was obtained.

The solubility of the obtained rubidium perrhenate in selected organic solvents such as xylene, acetone, acetonitrile, ethanol, dimethylformamide (DMF), isopropanol, and dimethyl sulfoxide (DMSO) was analyzed. The following procedure was used: 0.1 g RbReO_4_ was dissolved in 0.05 dm^3^ of the selected solvent and vigorously mixed for 30 min.

The thermal stability of the prepared rubidium perrhenate was also analyzed: 0.5 g of the material was dried at the selected temperature to constant mass. The temperature range of 40–180 °C was examined. Additionally, thermalgravimetric analysis was performed to examine the stability over a wider temperature range, from 30 to 1000 °C, using a heating rate of 10 °C/min under an argon atmosphere (volumetric flow rate: 150 cm^3^/min).

### 2.3. Characterization

IMN’s Department of Analytical Chemistry was responsible for all of the necessary analysis. The rhenium and rubidium contents in the product were analyzed using a weight method with tetraphenylarsonium chloride (TPAC) as the precipitating agent and flame atomic emission spectroscopy (FAES, spectrophotometer AAS novAA400, (Persee, Auburn, Canada), respectively. Analysis of the most important contaminants, i.e., As, Bi, Mo, Na, Ni, and Pb, was performed using ICP-MS (inductively coupled plasma mass spectrometry, ICP MS NexION, PerkinElmer, Waltham, MA, USA), while Ca, Cu, Fe, K, Mg, and Zn were assessed by ICP-OES (inductively coupled plasma optical emission spectrometer, ULTIMA 2, Horiba Jobin-Ivon, Kyoto, Japan). Solutions were analyzed by FAAS (flame atomic absorption spectroscopy, SOLAAR S4, THERMO, Waltham, MA, USA) equipped with a flame module and deuterium background correction to establish the rhenium and rubidium contents. X-ray powder diffraction analysis was carried out using Co Kα radiation in the 2*θ* range of 10°–100° (XRD 7, Seifert-FPM, Freiberg, Germany). This allowed us to calculate the crystallite size using the Scherrer method assuming no strain:

(6) $$L=\frac{K\lambda }{B\left(2\theta \right)cos\theta }$$

where *L* is the crystallite size, *K* is the shape factor ca. 0.9 (spherical crystallites), *λ* is the X-ray wavelength, *θ* is Bragg’s angle, and *B*(2*θ*) is the line-broadening at half-maximum. The validity of this technique has been compared with other methods by other authors [22]. Thermal properties were evaluated using a moisture analyzer (WPS 210S, Mettler Tolledo, Columbus, OH, USA) and thermogravimetric analyzer (STA 409 C/CD, Netzch, Selb, Germany).

## 3. Results and Discussion

### 3.1. Selection of Ion-Exchange Resin—Sorption Stage

For the initial investigation four ion-exchange resins (polystyrene crosslinked with divinylbenzene) were selected. Three of them were microporous (SP112, CT169, C160) with higher thermal stability according to data sheets, while the last one was a gel resin (PFC100X10).

Based on the preliminary experiments it was determined that the efficiency of rubidium ion sorption ranged from 67.8% for SP112 to 95.0% for PFC100X10. The highest Rb^+^ sorption efficiency was obtained for gel ionite PFC 100X10. This resin has a particle size of 570 ± 50 µm. For the investigated microporous resins, the highest sorption efficiency was obtained for ionite C160 (particle size between 300–1200 µm). No influence of the particle size on the Rb^+^ sorption efficiency was observed.

The concentration of rubidium in the solution after sorption is another important parameter. For the three investigated resins, it was 0.2–0.5 g/dm^3^.

Due to its low Rb^+^ sorption efficiency, ionite SP112 was eliminated from further investigation. Detailed data are presented in Table 2.

### 3.2. Effect of Temperature on the Rubidium Sorption Efficiency

**Figure 1:**
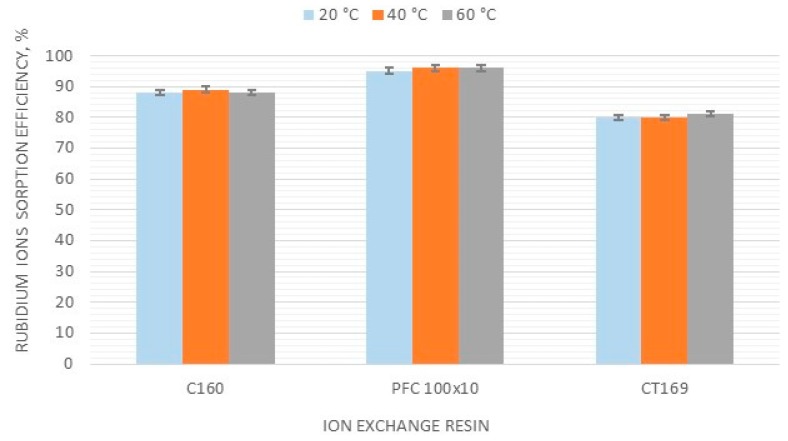
Effect of temperature on the rubidium ion sorption efficiency.

Similar results were observed for rubidium sorption tests at different temperatures. The highest efficiency in the whole analyzed temperature range was noticed for PFC 100x10 ionite; this was 95% at 40 and 60 °C. Consequently, this material was proven to have the highest affinity to monovalent rubidium ions. The efficiency of C160 resin within the examined temperature range was stable at around 90%, while for CT169, it was around 80%. The influence of temperature on the rubidium sorption efficiency for the three selected resins is presented in Figure 1.

No significant effect of temperature on sorption efficiency was observed for any of the three resins. Research was then continued at room temperature.

### 3.3. Effect of Contact Time on Rubidium Sorption Efficiency

**Figure 2:**
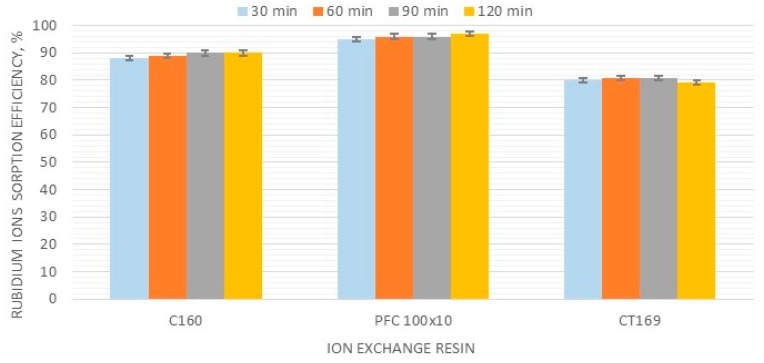
Effect of contact time on the rubidium ion sorption efficiency.

The examined contact times of the sorption process were in the range of 30–120 min. The best results were found for PFC100x10. The sorption efficiency for this resin was 92% and 98% after 30 and 120 min respectively. An increase in efficiency, from 88% to 90%, with the contact time was also observed for C160 resin, while C169 resin had a stable sorption efficiency of around 80%. However, this value was the lowest of the three examined resins. The results of these tests for the three selected ionites are shown in Figure 2.

It was suggested that a contact time of 60 min is the most appropriate for further research. Additionally, based on the low efficiency data obtained for C169, it was excluded from further testing.

### 3.4. Effect of the Rubidium Concentration on Rubidium Sorption Efficiency

The initial rubidium concentration of a solution directed to undergo sorption is an important parameter, which has an influence on the sorption efficiency. These tests allowed us to determine not only the sorption efficiency, but also the maximum degree of resin saturation with Rb^+^ ions as well as the limited initial concentration of rubidium sent to sorption. The results presented in Table 3 indicate that ca. 90% sorption efficiency was obtained for C160 ionite when the initial Rb concentration was 5.0 g/dm^3^. In the case of PFC100x10 resin, a significant increase in the sorption efficiency (to 80%) was observed for the initial Rb concentration (>2.0 g/dm^3^). The maximum degree of ionite saturation was similarly high for C160 and PFC100X10 resins, about 4.6%.

Over the whole investigated range, the rubidium saturation degree increased with the initial rubidium concentration for both resins. It was in the ranges 0.02–4.64% and 0.03–4.67% for C160 and PFC100X10, respectively. A similar effect was observed for the rubidium sorption efficiency, which was in the ranges 12.0–92.7% and 26.8–98.0% for C160 and PFC100X10, respectively. Therefore, both ionites were selected for further investigation.

### 3.5. Selection of Ion-Exchange Resin—Elution Stage

A big difference in rubidium ion elution using perrhenic acid was found in the first tests of both resins. This value was as high as 93.2% for PFC100x10 and was merely 40.5% for C160. The test results are shown in Table 4.

A high rubidium elution efficiency was observed for PFC100X10. Moreover, this resin showed high stability and enhanced sorption parameters (Table 2 and Table 3). Therefore, PFC100X10 was chosen for the rubidium ion elution tests.

### 3.6. Effect of Contact Time on the Rubidium Elution Efficiency

It was observed that an increase in the contact time between the eluent and rubidium-sorbed PFC100x10 resin from 30 to 180 min slightly improved the Rb^+^ sorption efficiency from 93.2% to 99.3%. The results of the tests are shown in Table 5.

For further investigation (dynamic tests), a contact time of 120 min was selected as the optimum one. This was chosen by the rubidium concentration in the solution after elution, which was more than 9.0 g/dm^3^.

### 3.7. Effect of Temperature on the Rubidium Elution Efficiency

It was observed that an increase in the elution process temperature decreased the Rb^+^ elution efficiency from 99.3% to 93.8%. The results are presented in Table 6.

Therefore, due to efficiency and economic reasons (energy saving), as well as to reduce the equipment issues that accompany the dynamic conditions of the process, elution under room temperature was selected.

### 3.8. Influence of the Rhenium-To-Rubidium Ratio on Rubidium Elution Efficiency

The increase in the Re:Rb ratio improved the Rb^+^ elution efficiency. For a ratio of 5:1, the Rb^+^ elution efficiency was only 25.1%. For a ratio of 10:1, the elution efficiency increased to >99%. The results of the tests are shown in Table 7.

For further investigation (dynamic tests), the optimum Re:Rb ratio was selected. It has to be pointed out this value was selected not only based on high elution efficiency of rubidium ions (99.3%), but mainly because it produced the highest rubidium concentration in solution after elution. Consequently, for Re:Rb ratios of 5:1 and 20:1, similar rubidium concentrations in eluates were achieved, 4.6 and 5.2 g/dm^3^, respectively. However, for a Re:Rb ratio of 10:1, it almost doubled to 9.5 g/dm^3^ Rb. This phenomenon (increase in Re:Rb ratio that improved Rb^+^ elution efficiency) can be explained by the rubidium ion elution being satisfied by a proper concentration of hydronium ions with appropriately high mobility. This can be achieved by excess added rhenium. Perrhenic acid is not a typical eluate in comparison to sulfuric acid, hydrochloric acid, and nitric acid. The application of acid with such a big anion size (to elution) may deteriorate the cation mobility. Consequently, this may change the ion exchange of sorbed rubidium ions with hydronium ions from the eluting solution.

### 3.9. Analysis of Three Cycles of PFC100x10 under Dynamic Conditions

**Figure 3:**
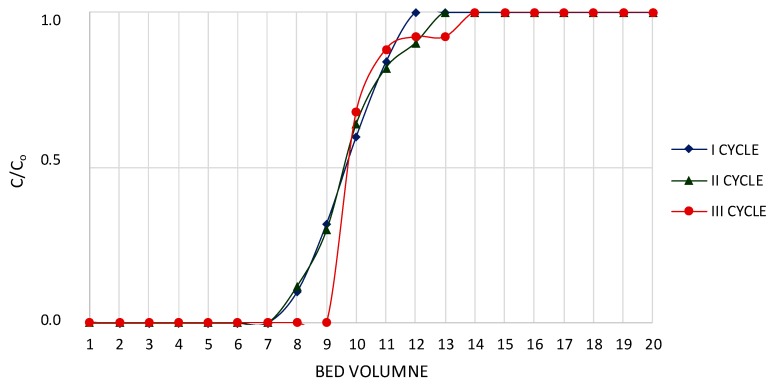
Characteristics of PFC100x10 during sorption.

**Figure 4:**
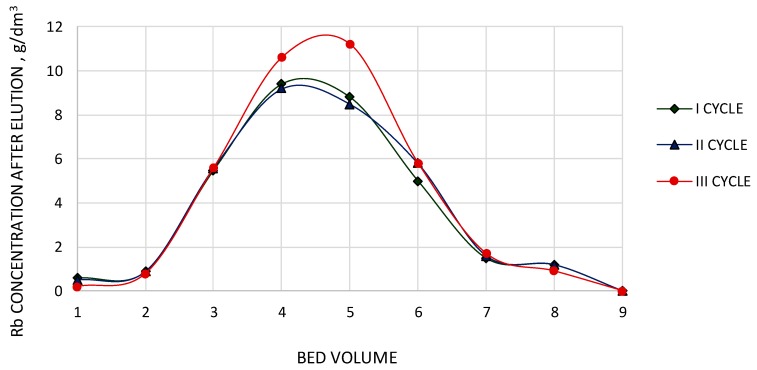
Characteristics of PFC100x10 during elution.

Based on sorption and elution tests, PFC100x10 was marked as the most appropriate and promising resin for rubidium sorption efficiency. Dynamic tests were composed of three operation cycles using PFC100x10. The results of the sorption tests are shown in Figure 3 and Table 8, while the results of the elution tests are shown in Figure 4 and Table 9. A sorption efficiency as high as 72.7–75.4% and a 4.5–4.9% degree of resin saturation by Rb^+^ were obtained. In all cycles, the elution efficiency was above 70%. A small increase in the elution efficiency in subsequent cycles was noticed, indicating that the ion-exchange resin operated correctly. The results including RbReO_4_ crystallization tests and impurity contents in crystallized RbReO_4_ are shown in Table 10 and Table 11, respectively, while the impurity content of crystallized RbReO_4_ after purification is shown in Table 12.

BV_1_ is the bed volume for which the rubidium concentration is <0.01 g/dm^3^; BV_2_ is the bed volume for a totally filled column; BV_3_ is the bed volume for which the rubidium concentration is >5.0 g/dm^3^ at elution; and BV_4_ is the bed volume for which the rubidium concentration is >1.0 g/dm^3^ at elution and at washing after elution.

The stable behavior of ion-exchange resin during sorption is presented in Figure 3. Three steep curves were obtained in all three cycles. This allowed us to distinguish between specific stages of sorption, which enabled us to increase the process efficiency on the industrial scale by using a proper process distribution. It was observed that the amount of solution for which the rubidium concentration was <0.01 g/dm^3^ (intentional) in subsequent cycles significantly increased from 6 BV (cycle I) up to 9 BV (cycle III). The rubidium efficiency was stable for all three cycles and was as high as 70% (Table 8). The parameter that was important to satisfy proper sorption was the ion-exchange saturation degree, with rubidium ions oscillating around 5 (Table 8). This parameter indicates a high affinity of resin to rubidium ion sorption.

All elution curves presented in Figure 4 are appropriately steep and indicate a proper run of the elution process. For all cycles, the maximum rubidium concentration of eluates was in the range 8–12 g/dm^3^, which allowed rubidium perrhenate to crystallize. The increase in concentration in the subsequent cycles of resin processing was favorable. The elution efficiency was high (70%) and stable, as presented in Table 9.

### 3.10. Crystallization and Purification Stages

Solutions of different rubidium concentrations, i.e., 1.0, 3.0, 6.0 and 9.0 g/dm^3^, were prepared for crystallization investigations. The results of these tests are shown in Table 10.

It was noticed that the higher concentrations of rubidium were associated with a higher crystallization efficiency, as shown in Table 10. It was found that it is impossible to crystallize rubidium perrhenate using solution with a concentration of <1.0 g/dm^3^ Rb, while above 3.0 g/dm^3^ Rb, it was possible to crystallize products with a stochiometric rubidium and rhenium ratio. When the Rb concentration was around 9.0 g/dm^3^, a product could be obtained with an efficiency of as high as 90%.

Additionally, with an increase in the concentration of rubidium, the crystallite size of crystallized products was diminished. Under these conditions, it was possible to produce rubidium perrhenate with a contaminant content below 200 ppm (Table 11), while after purification, using H_2_O_2_ solution and acetone, metallic contamination may drop to 21 ppm (Table 12).

**Figure 5:**
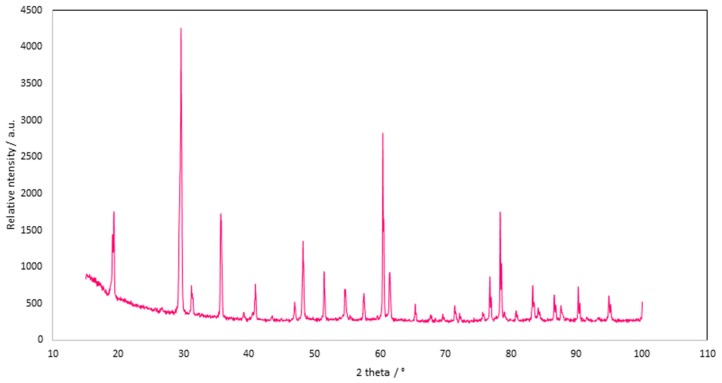
XRD pattern of rubidium perrhenate crystals.

An efficiency of crystallized rubidium perrhenate of over 88% may be obtained if the concentration of rubidium in the initial solution exceeds 9.0 g/dm^3^ Rb. Application of double purification i.e., using 0.005 dm^3^ 10% H_2_O_2_ solution (to remove iron and nickel) and subsequent washing with 0.02 dm^3^ acetone (to remove magnesium, sodium, and zinc) enabled rubidium perrhenate to be obtained. The obtained material was characterized by a low impurity content: <2 ppm As, <2 ppm Bi, <5 ppm Ca, <5 ppm Cu, <3 ppm Fe, <10 ppm K, <3 ppm Mg, <5 ppm Mo, <2 ppm Na, <5 ppm Pb, and <3 ppm Zn) and stoichiometric ratios of Rb and Re of 22.5 wt.% and 55.4 wt.%, respectively. The XRD analysis (presented in Figure 5) confirmed that pure rubidium perrhenate was obtained [22]. Additionally, it was found that RbReO_4_ was soluble in dimethylformamide and dimethyl sulfoxide, sparingly soluble in acetonitrile, and insoluble in other solvents (xylene, acetone, ethanol, and isopropanol).

### 3.11. Thermal Stability

**Figure 6:**
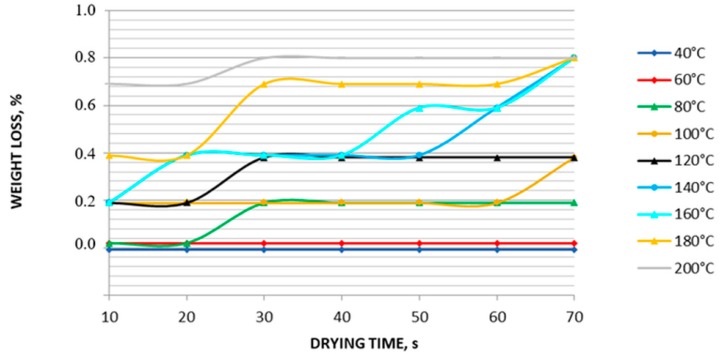
Drying stability of rubidium perrhenate at 40–200 °C.

**Figure 7:**
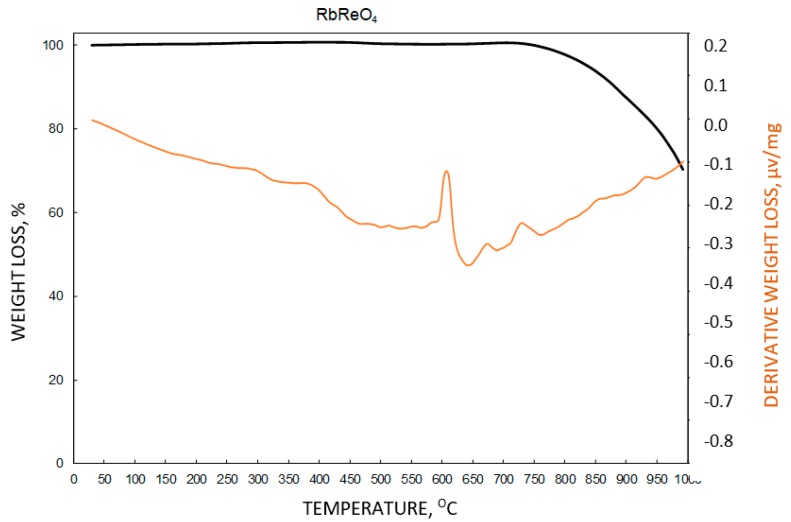
Drying stability of rubidium perrhenate at 30–1000 °C.

The results obtained using thermobalance showed that RbReO_4_ was thermally stable in the temperature range 40–200 °C (Figure 6). The maximum mass loss, close to 1%, was observed at 140–200 °C. Therefore, temperatures below 140 °C were considered as the most appropriate for RbReO_4_ drying. The results are presented in Figure 6. Analysis of a wider temperature range showed that rubidium perrhenate decomposes at 733–995 °C, which means that it is thermally stable (Figure 7).

Rubidium perrhenate was stable at up to 730 °C, and then it started to decompose. This mass loss was observed as a single step which was attributed to rhenium oxide formation—both Re_2_O_7_ and ReO_3_. Both reactions were observed in a similar temperature range.

### 3.12. Developed Technology Scheme

Based on the experimental results, it was possible to develop a technological scheme for the production of high-purity rubidium perrhenate. The proposed technology was also successfully patented [23]. It is composed of 10-unit operations. In the first step, aqueous rubidium nitrate solutions were subjected to rubidium sorption on PFC100x10 ion-exchange resin in hydrogen form. Six cycles of rubidium perrhenate preparation were also performed under dynamic conditions on a large-scale. The bed (1 kg of PFC100X10 ionite) is placed inside a column, with a height to diameter ratio of over eight. Sorption wascarried out downwards of the column at room temperature using RbNO_3_ solution (4.5 g/dm^3^ Rb) until the concentration of rubidium in the effluent achieves the initial level. The contact time between the solution and bed was up to 60 min. Then, demineralized water was used to wash the ion-exchange resin. The stream of post-sorption and post-washing solutions is combined and used for the preparation of the initial solution. Rubidium was eluted from the washed bed using a solution of perrhenic acid (500 g/dm^3^ Re) at ambient temperature. The contact time between the eluent (HReO_4_) and resin was at least 120 min. The eluate was divided into two parts. The first one, containing less than 2.0 g/dm^3^ Rb, was recycled to prepare a solution for elution, while the second (main) one was concentrated and crystallized.

**Figure 8:**
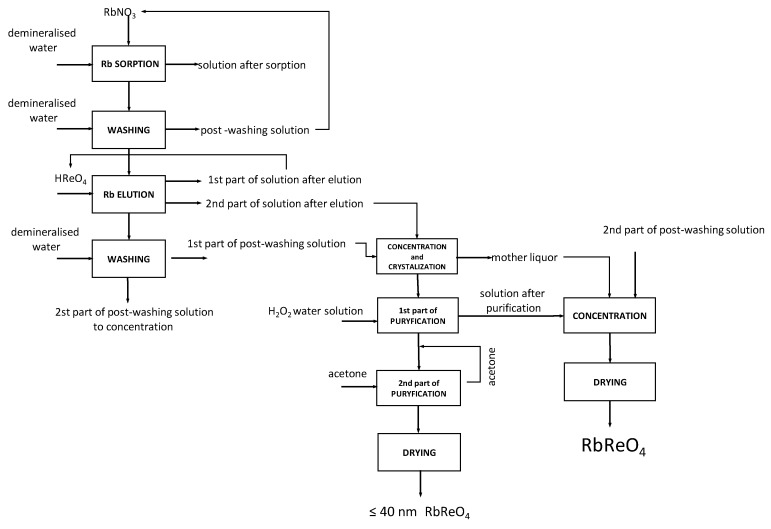
Technological scheme of anhydrous rubidium perrhenate preparation.

The post-washing solutions from elution were divided into two parts—the first was combined with the solution subjected to concentration, while the other, was mixed with mother liquors and post-purification solutions and then sent for rubidium perrhenate preparation (>40 nm). The solution produced in the second part of the elution (containing >9.0 g/dm^3^ Rb) was concentrated and crystallized. The temperature of the concentration process should preferably not exceed 80 °C, and vigorous stirring is suggested. After quenching, the resulting rubidium perrhenate was crystallized. Obtained crystals were filtered and subjected to two-stage washing, first with 10% H_2_O_2_ solution, followed by anhydrous acetone. In the last step, the obtained crystals of rubidium perrhenate were dried to a constant mass at 140 °C, and heated at 400 °C. This method allows anhydrous nanocrystalline (≤40 nm) RbReO_4_ with a stoichiometric composition to be prepared in a reproducible way. Other waste solutions, i.e., the second part from washing after elution, mother liquors, and aqueous washings, were used for concentration (up to 80 °C) and rubidium perrhenate extraction with a contamination level below 1.0%. The obtained crystals of rubidium perrhenate were dried to constant mass at 140 °C. In Figure 8, the technological scheme of anhydrous nanocrystalline rubidium perrhenate preparation is presented.

**Figure 9:**
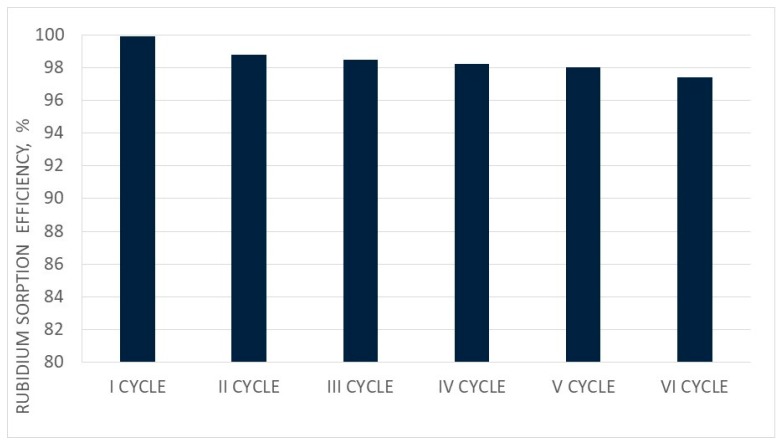
Changes in sorption efficiency in subsequent cycles (I–VI).

**Figure 10:**
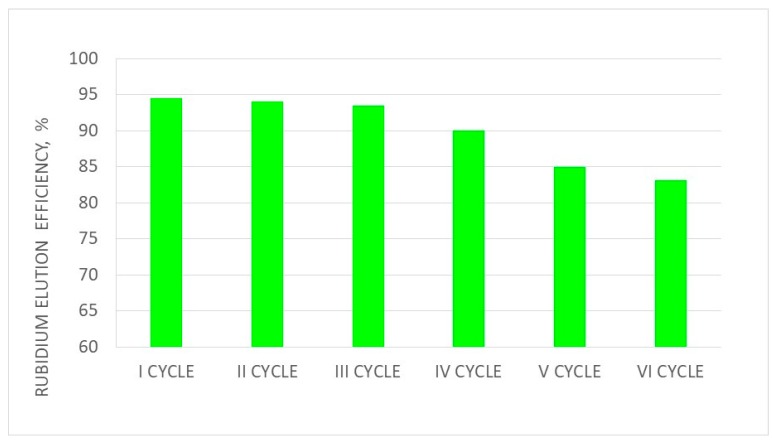
Changes in the elution efficiency in subsequent cycles (I–VI).

**Figure 11:**
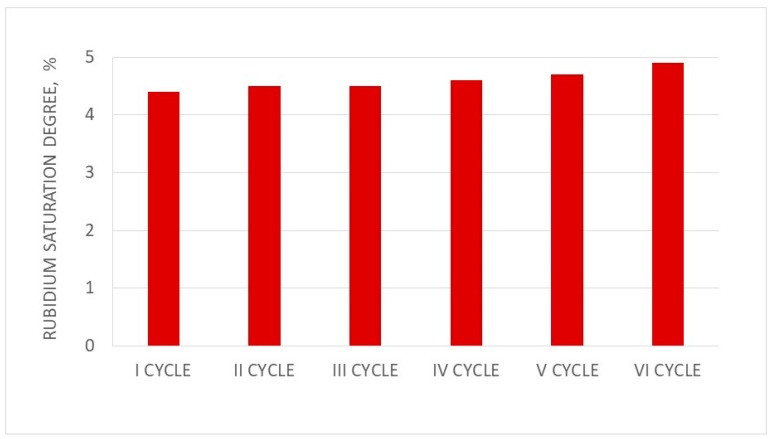
Changes in the saturation degree of resin by Rb in subsequent cycles (I–VI).

The mass balances of rubidium and rhenium sorption and elution for the first and sixth cycles are presented in Table 13 and Table 14. Changes in sorption and elution efficiencies as well as the saturation of resin by Rb in subsequent cycles of ionite work are presented in Figure 9, Figure 10 and Figure 11, respectively.

The sorption efficiency of Rb ions in six subsequent cycles of ionite (PFC100X10) was very high (99.93–97.39%), but it slowly decreased in subsequent cycles. The saturation degree of PFC 100X10 ionite with Rb ions was 4.4–5.7%. It has to be emphasized that the rubidium concentration was low in solutions after sorption, around 0.01–0.1 g/dm^3^, and the rubidium concentration values in solutions after washing were in the same range. These solutions were mixed and used in the next portion of rubidium nitrate dissolution—eliminating rubidium losses. Appropriate eluate division regarding the Rb concentration allowed the two types of rubidium perrhenate to crystallize, producing nanocrystalline rubidium perrhenate (<40 nm) from solutions with a Rb concentration above 9.0 g/dm^3^, and amorphous rubidium perrhenate. Both products can be treated as good catalysts precursors, although their purity may vary. The nanocrystalline material is of a high purity with a contaminant content of <21 ppm, and while amorphous may contain up to 1% contaminants. Proper management of waste solutions from sorption, elution, and washing make this technology practically waste-free with minimized rhenium and rubidium losses in all ten cycles at a level of <0.1%. Additionally, the readiness level of this technology is high as it was tested in the normal laboratory, large laboratory, and at the pilot scale. Rhenium and rubidium losses can be minimized, producing two products. This also enables the big excess of used rhenium and rubidium to be managed. The above results confirm that rubidium perrhenate production by ion exchange methods is a stable process.

## 4. Conclusions

It was determined that high-purity anhydrous rubidium perrhenate may be produced by the ion-exchange method using strongly acidic cation-exchange resin (PFC100x10). The crystallite size of the obtained material was smaller than 40 nm. Its purity met even the strictest requirements for catalyst production, with a contaminant content as low as <2 ppm As, <2 ppm Bi, <5 ppm Ca, <5 ppm Cu, <3 ppm Fe, <10 ppm K, <3 ppm Mg, <5 ppm Mo, <2 ppm Na, <5ppm Pb, and <3 ppm Zn. Stoichiometric amounts of rubidium and rhenium, 22.5 wt.% and 55.4 wt.%, respectively, were achieved. The developed method allowed rubidium perrhenate to be produced from the remaining waste solutions. The material contained 22.4 wt.% Rb and 55.3 wt.% Re and a metal contaminant content below 1.0%. Consequently, this technology minimizes rubidium and rhenium losses.

## Figures and Tables

**Table 1:** Properties of selected strongly acidic cation-exchange resins.

Resin	Structure	Functional Group	Moisture Retention %	Particle Size Range µm
C160	macroporous polystyrene crosslinked with divinylbenzene	sulfonic acid	35–40	300–1200
SP112			56–60	670 ± 50
CT169			51–57	425–1200
PFC100x10	gel polystyrene crosslinked with divinylbenzene		44–48	570 ± 50

**Table 2:** Results of ion-exchange resin selection for rubidium sorption.

Ion-Exchange Resin	Volume of a Solution after Sorption dm^3^	Rb Concentration in a Solution after Sorption g/dm^3^	Rb^+^ Sorption Efficiency %
SP112	0.115	1.4	67.8 ± 0.9
PFC100	0.125	0.2	95.0 ± 0.3
CT169	0.120	0.8	80.8 ± 0.8
C160	0.112	0.5	88.8 ± 0.7

**Table 3:** Effect of the rubidium concentration on the rubidium ion sorption efficiency.

Ion-Exchange Resin	Rb Concentration g/dm^3^	Volume of a Solution after Sorption dm^3^	Rb Concentration in a Solution after Sorption g/dm^3^	Rb^+^ Sorption Efficiency %	Rb Saturation Degree %
C160	0.5	0.110	0.4	12.0 ± 0.2	0.02 ± 0.01
	1.0	0.110	0.6	34.0 ± 0.2	0.09 ± 0.01
	2.0	0.121	0.6	63.7 ± 0.3	0.32 ± 0.02
	5.0	0.112	0.5	88.8 ± 0.2	1.11 ± 0.02
	10.0	0.112	0.9	89.9 ± 0.3	2.25 ± 0.03
	15.0	0.110	1.1	91.9 ± 0.3	3.45 ± 0.02
	20.0	0.112	1.3	92.7 ± 0.4	4.64 ± 0.03
PFC100x10	0.5	0.122	0.3	26.8 ± 0.2	0.03 ± 0.01
	1.0	0.120	0.3	64.0 ± 0.2	0.16 ± 0.01
	2.0	0.120	0.3	82.0 ± 0.2	0.41 ± 0.02
	5.0	0.125	0.2	95.0 ± 0.3	1.19 ± 0.02
	10.0	0.121	0.8	96.3 ± 0.4	2.26 ± 0.02
	15.0	0.123	1.0	97.0 ± 0.4	3.44 ± 0.03
	20.0	0.121	1.1	98.0 ± 0.3	4.67 ± 0.02

**Table 4:** Results of ion-exchange resin selection for rubidium sorption.

Ion-Exchange Resin	Volume of a Solution dm^3^	Rb Concentration in a Solution after Elution g/dm^3^	Rb^+^ Elution Efficiency %
C160	0.025	3.4	40.5 ± 0.7
PFC100x10	0.025	8.2	93.2 ± 0.9

**Table 5:** Effect of the contact time on the rubidium ion elution efficiency.

Ion-Exchange Resin	Contact Time min	Volume of a Solution dm^3^	Rb Concentration in a Solution after Elution g/dm^3^	Rb^+^ Elution Efficiency %
PFC100x10	30	0.025	8.2	93.2 ± 0.8
	60	0.024	8.9	97.1 ± 0.9
	120	0.023	9.5	99.3 ± 0.8
	180	0.024	9.1	99.3 ± 0.7

**Table 6:** Effect of temperature on the rubidium ion elution efficiency.

Ion-Exchange Resin	Temperature °C	Volume of a Solution dm^3^	Rb Concentration in a Solution after Elution g/dm^3^	Rb^+^ Elution Efficiency %
PFC100x10	20	0.023	9.5	99.3 ± 0.8
	40	0.022	9.3	93.0 ± 0.9
	60	0.024	8.6	93.8 ± 0.9

**Table 7:** Effect of the rhenium-to-rubidium ratio on the rubidium ion elution efficiency.

Ion-Exchange Resin	Re:Rb Ratio	Volume of a Solution dm^3^	Rb Concentration in a Solution after Elution g/dm^3^	Rb^+^ Elution Efficiency %
PFC100x10	5:1	0.012	4.6	25.1 ± 0.8
	10:1	0.023	9.5	99.3 ± 0.8
	20:1	0.042	5.2	99.3 ± 0.01

**Table 8:** Parameters for rubidium ion sorption—PFC100x10.

Cycle No.	BV_1_	BV_2_	Rb^+^ Sorption Efficiency %	Rb Saturation Degree %
I	6	13	75.2	4.5
II	7	13	72.7	4.7
III	9	14	75.4	4.9

**Table 9:** Parameters for rubidium ion elution—PFC100x10.

Cycle No.	BV_3_	BV_4_	Rb^+^ Elution Efficiency %
I	4	6	72.5
II	4	6	72.5
III	4	6	79.8

**Table 10:** Results of the RbReO_4_ crystallization tests.

Test No.	*c*^0^_KRb_ g/dm^3^	*c*^0^_KRe_ g/dm^3^	Crystallite Size nm	Crystals Mass g	*X*_Rb_ %	*X*_Re_ %	*W*_KRbReO4_ %
1	1.0	56.0	*	0.5	*	*	
2	3.0	74.0	39	2.5	22.5	55.3	55.6
3	6.0	82.0	30	6.0	22.4	55.4	66.7
4	9.0	99.0	25	12.0	22.5	55.4	88.9

*: analysis not performed due to small mass of crystals; c^0^_KRb_: rubidium concentration in a solution sent to crystallization; c^0^_KRe_: rhenium concentration in a solution for crystallization; X_Rb_: content of rubidium in crystals; X_Re_: content of rhenium in crystals; W_KCRbReO4_: efficiency of crystallization (i.e., mass ratio of crystals to theoretical one calculated based on the content of rubidium in a solution).

**Table 11:** Impurity contents in crystallized RbReO_4_.

Test No.	As	Bi	Ca	Cu	Fe	K	Mg	Mo	Na	Ni	Pb	Zn
	ppm											
3	<2	<2	<5	<5	17	<10	22	<5	55	28	<5	21
4					15		25		50	26		20

**Table 12:** Impurity contents in crystallized RbReO_4_ after purification.

Purification Tests	Fe	Mg	Na	Ni	Zn
	ppm				
no purification	15	25	50	26	20
5% H_2_O_2_	5	10	20	21	15
10% H_2_O_2_	<3	10	10	<10	12
acetone	6	5	5	26	5
10% H_2_O_2_ + acetone	<3	<3	<2	<10	<3

**Table 13:** Rubidium and rhenium sorption and elution mass balance—cycle I.

Materials		Solution Volume dm^3^	Rb Concentration g/dm^3^	Rb Mass g	Re Concentration g/dm^3^	Re Mass g	Process Efficiency %	Rubidium Saturation Degree %
To Sorption	solution	10.0	4.50	45.00	-	-	-	
	washing water	2.5	-	-	-	-	-	
After Sorption	solution	10.2	0.10	1.13			99.93	4.5
	washing	2.3	0.08	0.19				
To Elution	Ionite (1 kg)			44.97	-	-	-	-
	eluent	2.0	500.00	-	-		-	-
	washing water	3.0	-	-				
After Elution	elution (1st part)	1.0	1.66	1.66	1.58	1.58	-	-
	elution (2nd part)	2.0	17.90	35.80	468.00	936.00	94.51	-
	washing (1st part)	1.0	6.70	6.70	54.10	54.10		
	washing (2nd part)	2.0	0.40	0.80	4.16	8.32	-	-

**Table 14:** Rubidium and rhenium sorption and elution mass balance—cycle VI.

Materials		Solution Volume dm^3^	Rb Concentration g/dm^3^	Rb Mass g	Re Concentration g/dm^3^	Re Mass g	Process Efficiency %	Rubidium Saturation Degree %
To Sorption	solution	11.2	4.52	50.62	-	-	-	
	washing water	2.5	-	-	-	-	-	
After Sorption	solution	11.3	0.05	0.070			97.39	5.7
	washing	2.4	0.01	0.017				
To Elution	ionite (1 kg)			49.30	-	-	-	-
	eluent	2.0	500.00	-	-		-	-
	washing water	3.0	-	-	-	-	-	-
After Elution	Elution (1st part)	1.0	3.20	3.20	1.67	1.67		
	Elution (2nd part)	2.0	14.0	28.0	468.00	936.00	83.16	-
	Washing (1st part)	1.0	13.0	12.00	53.30	53.30		
	Washing (2nd part)	2.0	6.3	12.6	4.50	9.00		
